# Radiation Recall Pneumonitis After Treatment With Checkpoint Blockade Immunotherapy: A Case Series and Review of Literature

**DOI:** 10.3389/fonc.2021.662954

**Published:** 2021-04-30

**Authors:** Paul Riviere, Whitney Sumner, Mariel Cornell, Ajay Sandhu, James D. Murphy, Jona Hattangadi-Gluth, Andrew Bruggeman, Sangwoo S. Kim, J. Michael Randall, Andrew B. Sharabi

**Affiliations:** ^1^ Department of Radiation Medicine and Applied Sciences, University of California San Diego, La Jolla, CA, United States; ^2^ Divisions of Hematology-Oncology and Bone Marrow Transplantation, Moores Cancer Center, University of California San Diego, La Jolla, CA, United States

**Keywords:** radiation, immunotherapy, pneumonitis, reaction, checkpoint, PD-1

## Abstract

**Background:**

Radiation recall pneumonitis (RRP) is a poorly understood clinical syndrome in which patients develop radiation pneumonitis triggered by a systemic agent, often years after the completion of radiation therapy. Immune checkpoint blockade agents have only recently been posited as a trigger for RRP. Here, we present three cases of immunotherapy-induced RRP.

**Case Presentation:**

Our first patient was diagnosed with primary lung adenocarcinoma, and 4.5 years after completing radiation therapy developed symptomatic RRP immediately following a second dose of nivolumab-containing immunotherapy regimen. Our second patient was diagnosed with primary bladder cancer metastatic to the mediastinum, which was treated twice with radiation therapy. He developed RRP in the days following his second course of ipilimumab-pembrolizumab which was months after his second course of radiation that he received. Our final patient was diagnosed with metastatic small cell lung cancer and received local consolidative radiation therapy in addition to whole-brain radiation. He developed RRP on the 11^th^ day after concluding his 4^th^ cycle of nivolumab-ipilimumab, approximately 7 months after having had completed chest radiation therapy.

**Conclusions:**

Immunotherapy-induced RRP is a rare diagnosis which can present more focally than traditional immunotherapy pneumonitis and which must be clinically differentiated from other local processes such as pneumonia. Further research should explore the mechanisms underlying these radiation recall reactions as many patients receive radiation and immunotherapy during the course of their cancer treatment.

## Introduction

Radiation recall is a clinical phenomenon in which patients acutely develop signs and symptoms of inflammatory radiation toxicities or erythema within previously irradiated fields after initiation of a systemic therapy. Radiation recall typically arises after the timeframe during which any acute radiation toxicity would be expected (up to several years after completion of radiation therapy (RT)) and patients often tolerate re-challenge with the offending agent without recurrence of RRP (1). Numerous drugs have been linked to recall reactions, including cytotoxic antineoplastic medications, targeted cancer therapeutics, antibiotics, and even statins (2–4). While radiation recall has classically been described as a cutaneous reaction, more recently there has been increasing awareness of recall reactions occurring within other organ systems (2). One emerging diagnosis is radiation recall pneumonitis (RRP), a focal disease of lung parenchyma that clinically and radiographically resembles radiation pneumonitis, but which is temporally incongruent with radiation pneumonitis which typically occurs 1-3 months after conventional radiation (5). RRP has been described with chemotherapeutic agents (e.g. gemcitabine, doxorubicin, and docetaxel) (1, 6–8) and small-molecule kinase inhibitors (e.g. everolimus, sunitinib, erlotinib) (9–11). However, the literature is much sparser on RRP arising in the context of immunotherapy (12–14), with the largest case series containing only two patients (13). Here, we describe three cases of suspected immunotherapy-induced RRP treated at our institution.

## Case Descriptions

### Patient 1

Patient 1 64 y/o man with a 48 pack-year cigarette smoking history presented to an outside hospital with a chronic cough in 2012, which was refractory to antibiotic therapy. Imaging revealed an approximately 3.7x2.3 cm right-sided mass in the minor fissure with associated hypermetabolic hilar and mediastinal lymphadenopathy, as well as an ipsilateral pleural effusion which was pathologically negative. Biopsy *via* EUS diagnosed poorly differentiated adenocarcinoma, TTF-1 positive, CK 7, Napsin A positive, CK 20 negative, CK 5 negative, CD45 negative, EGFR wild-type, ALK wild-type, ROS1 wild-type. Staging imaging showed a right paratracheal lymph node measuring 3.2 cm, a subcarinal lymph node measuring 3.3 cm, and a right hilar lymph node 5.5 cm in maximal dimension, but no evidence of distant metastatic disease; he was thus diagnosed with a T2N2 stage IIIb non-small cell lung adenocarcinoma, per AJCC 7 criteria. He completed 4 cycles of carboplatin with pemetrexed, followed by concurrent cisplatin and radiation therapy (intensity-modulated RT (IMRT) 59.4 Gy in 33 fractions) completed 2013, with good radiographic response. Surveillance imaging detected multiple new parenchymal lung lesions, the largest of which was 1.1cm in the left upper lobe, as well as precarinal mediastinal adenopathy measuring 1.3x1.8cm. He was started on first line systemic therapy for advanced disease with pemetrexed, carboplatin, and bevacizumab. After progressing with bilateral pulmonary nodules on imaging he was enrolled on clinical trials, and in the ensuing years received: single-agent pembrolizumab (partial response, treated 11 months); single-agent nivolumab (progressed, treated 10 weeks); single-agent gemcitabine (partial response, treated 6 months); a second course of single-agent pembrolizumab (progressed, treated 1 month); single-agent docetaxel (partial response but discontinued due to toxicity, treated 5 months); and single-agent atezolizumab (progressed, treated 2 months). Throughout these courses of therapy, he developed metastatic disease to the left kidney, vertebral body, right sided ribs, and bilateral lung parenchyma.

**Figure 1:**
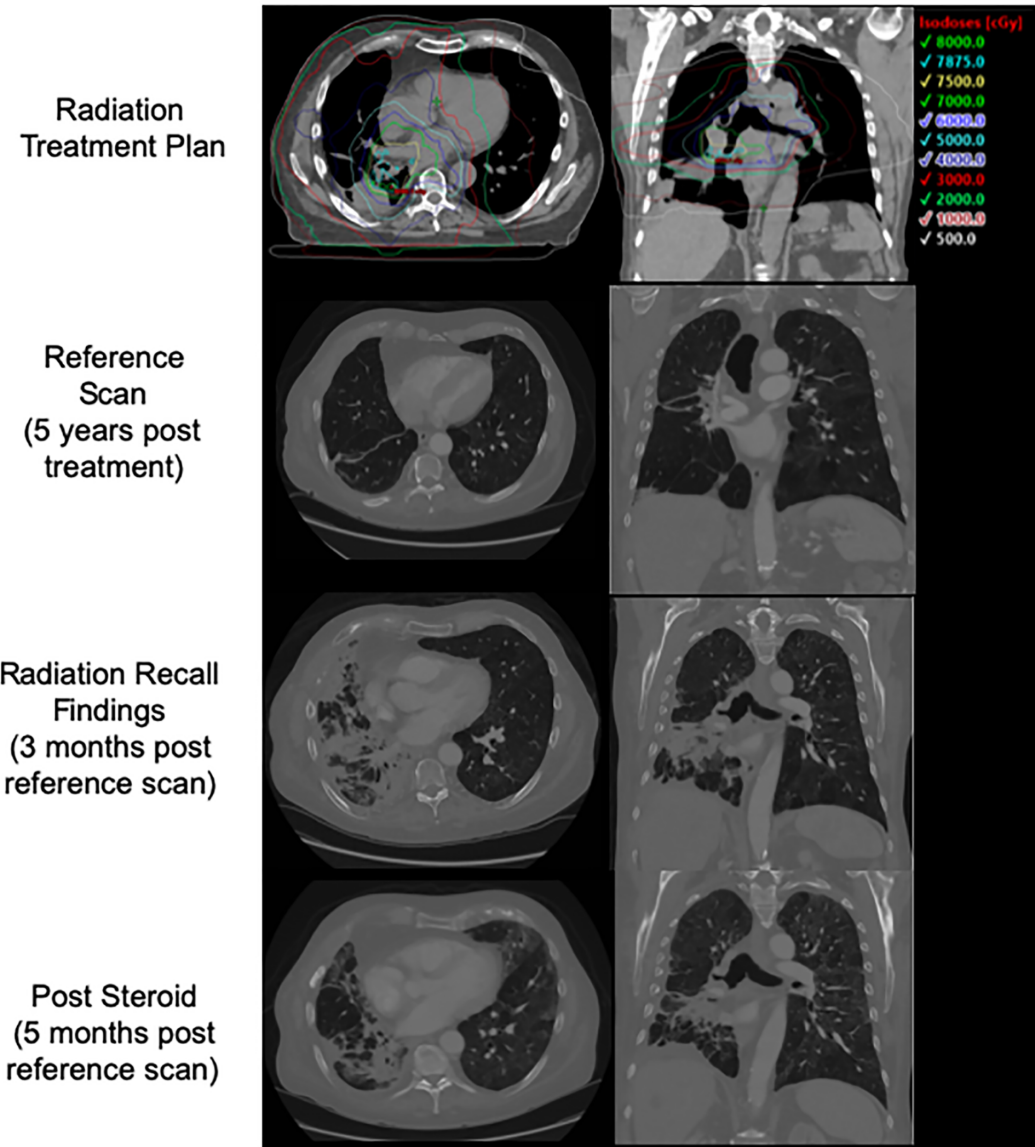
Patient 1's initial treatment plan, post treatment CT, CT at time of presentation with radiation recall, and CT following course of steroids with interval improvement.

In early 2018, he enrolled on a novel open-label immunotherapy trial, consisting of nivolumab combined with an experimental HDAC inhibitor. He received the first dose on 3/2018, and developed a cough approximately 2 weeks later, days following his second dose of the immunotherapy combination. After 2 weeks of symptomatic cough, on 5/2018 he started a course of empiric levofloxacin with no improvement. He completed his second cycle of therapy despite the cough becoming increasingly productive and having an increased home oxygen requirement. 2 weeks later, a CT of the chest with contrast revealed pneumonitis changes correlating to the previous IMRT fields (**Figure 1**), and was diagnosed with RRP. Cycle 3 was deferred and he started a tapered course of oral prednisone, 60 mg daily with excellent clinical response. After recovering, he tolerated a re-trial of monotherapy nivolumab (omitting the experimental agent) without a recurrent RRP. He has since received two courses of palliative radiation for chest wall lesions without complications.

### Patient 2

**Figure 2:**
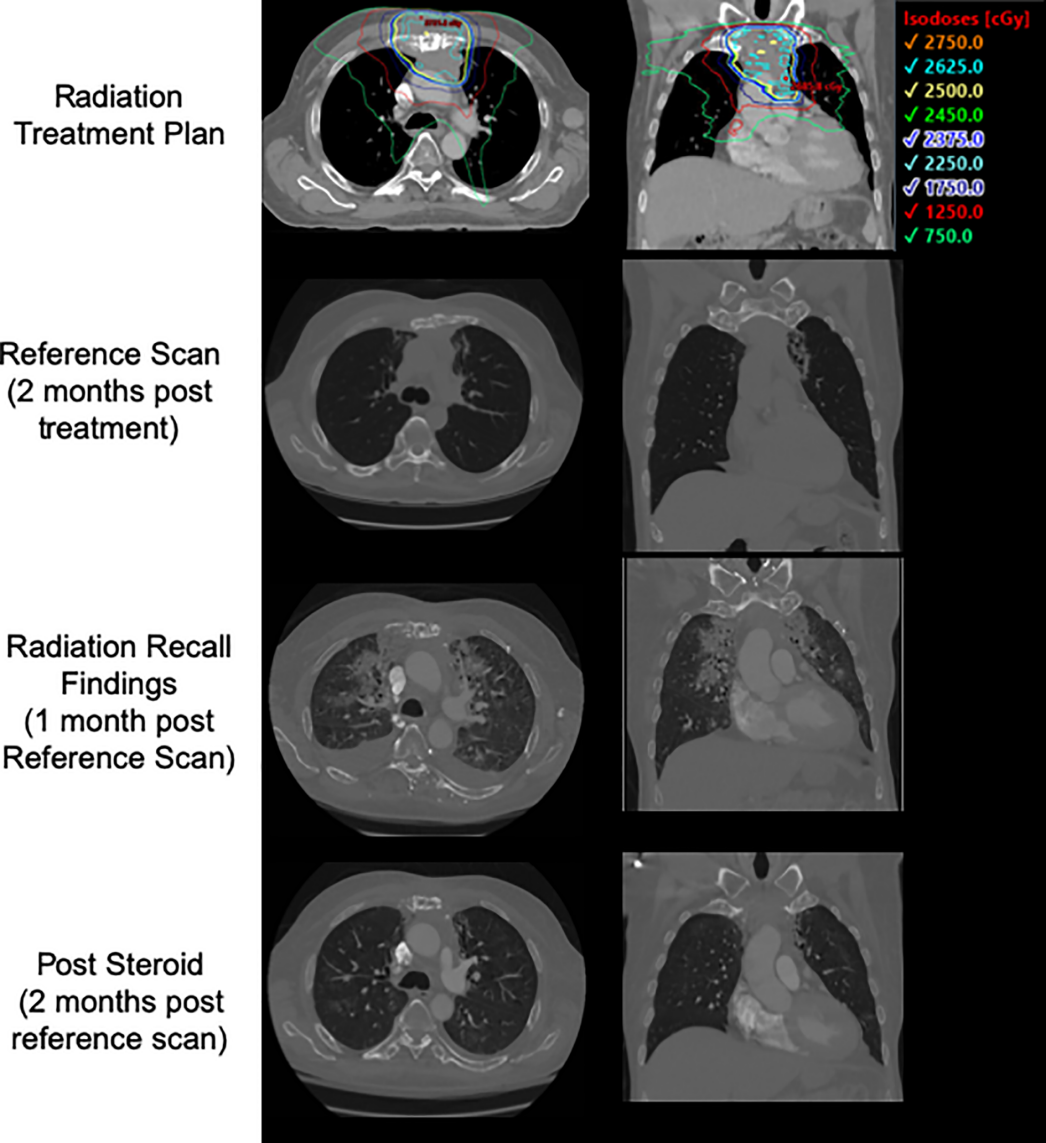
Patient 2's treatment plan, post treatment CT, CT at time of presentation with radiation recall, and CT following course of steroids with interval improvement.

Patient 2 originally presented to an outside hospital in 2015 complaining of 2 years of gross hematuria that had progressed to blood clots in his urine. At that time, he was 63 years old with a 20 pack-years cigarette smoking history, and had no other known risk factors for urogenital malignancies. He was diagnosed with a T2G3 urothelial cancer of the bladder dome with extensive invasion in the muscularis propria *via* transurethral resection of bladder tumor (TURBT). In mid 2015, and underwent a restaging TURBT which re-demonstrated high-grade urothelial carcinoma invading the muscularis propria, and CT chest revealed an anterior mediastinal mass that was positive for metastatic urothelial cancer. He completed frontline therapy with cisplatin and gemcitabine in 1/2016 (cisplatin exchanged for carboplatin after acute kidney injury during cycle 3), after which he received palliative-intent RT with 30 Gy in 10 fractions to the mediastinal mass, completed 4/2016. He then was treated with an experimental immunotherapeutic agent on trial combined with durvalumab and has a durable response over 3.5 years. He was noted to progress in his mediastinum and this site was retreated using SBRT to 25Gy in 5 fractions, completed in mid 2019. This was followed by monotherapy pembrolizumab as a bridge to a clinical trial for which he was ultimately deemed ineligible, and in late 2019, ipilimumab was added to his pembrolizumab regimen. 3 days following his second cycle of pembrolizumab-ipilimumab, he presented to the emergency room complaining of abdominal and flank pain. He was found to have peritonitis from his necrotic primary tumor, as well as radiographic evidence of RRP of the left lung (**Figure 2**). However, the patient reported no symptoms associated with this, and was breathing normally on room air. Unfortunately, his clinical status rapidly declined, and he expired 12 days later in the hospital.

### Patient 3

Patient 3 presented in 2017 at age 52 with headaches, and was subsequently diagnosed with lung cancer metastatic to the brain. He was found to have small-cell lung cancer from a left upper lobe primary tumor and underwent surgical resection of a 4.1 cm. metastasis in the left cerebellum *via* posterior fossa craniectomy. He completed a cycle of cisplatin/etoposide while hospitalized, and then underwent whole-brain radiation therapy to 37.5 Gy in 15 fractions (completed mid 2017), followed by 4 additional cycles cisplatin and etoposide (completed 2 months later), which was followed by consolidative conformal RT to the primary tumor and mediastinum to 30 Gy in 10 fractions, completed in mid 2017. The patient subsequently developed 4 new brain metastases, which were treated with SRS in late 2017, followed by ipilimumab with nivolumab.

**Figure 3:**
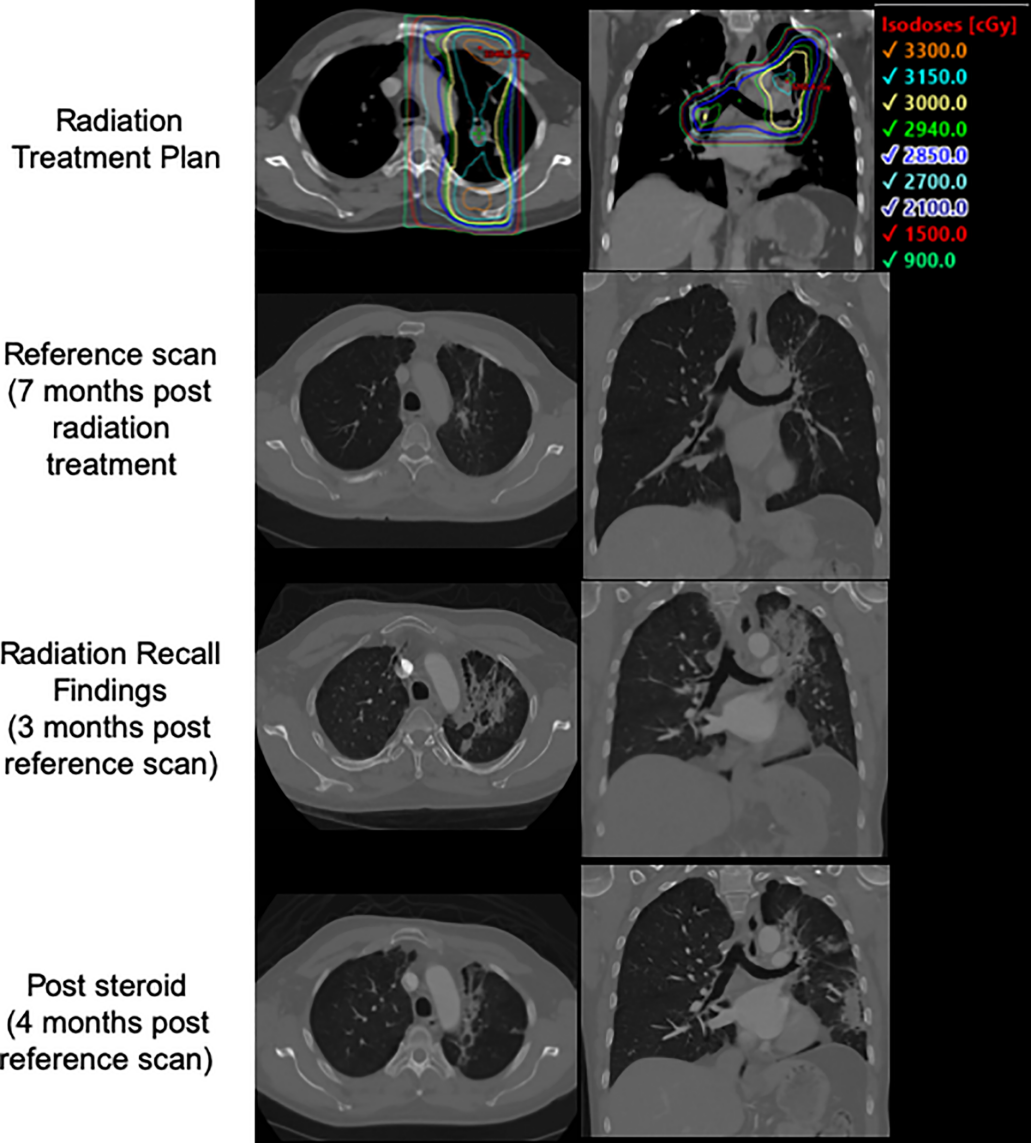
Patient 3's treatment plan, post treatment CT, CT at time of presentation with radiation recall, and CT following course of steroids with interval improvement.

Seven months after completing thoracic RT, 11 days after receiving his 4^th^ cycle infusion of nivolumab/ipilimumab, the patient developed acutely worsening left-sided chest pain, most severe upon inspiration and with no associated cough or fevers. He showed no infectious signs or symptoms, and was oxygenating well on room air. A CT scan of the thorax revealed evolving left lung fibrosis consistent with a recall reaction (**Figure 3**). Infectious workup was unrevealing, and the patient responded rapidly to a tapered course of oral prednisone 50 mg daily. Upon resolution of his symptoms his medical oncologists elected to halt systemic therapies without a re-challenge. To date he has received ablative radiotherapy to the left adrenal gland and SRS twice to new brain metastases without complication. 

## Conclusions

Immunotherapy-related pneumonitis is a well-documented (15, 16) adverse effect of immune checkpoint therapies. However, while prior RT may increase incidence of low grade pneumonitis in patients treated with these drugs (15, 17), radiologic manifestations of immunotherapy-related pneumonitis typically do not overlap with high-dose treatment fields (17), suggesting that RRP is indeed a distinct clinical entity. The pathophysiology of radiation recall remains an area of active investigation; authors have hypothesized radiation-induced: (1) sublethal stem cell damage/reprograming, in which surviving local stem cells lose future proliferative ability or develop aberrant inflammatory responses to systemic agents (2) hypersensitivity reaction in which radiation might lower the inflammatory response threshold, causing localized idiosyncratic drug reactions (3) changes to vascular permeability/proliferation causing local accumulation of systemic agents (i.e. pharmacokinetic effects), and (4) DNA damage and oxidative stress causing keratinocyte necrosis/depletion, amongst others (1, 6, 12, 18). Immunotherapy-mediated RRP implies that direct cytotoxic drugs may not be required to induce recall reactions. Additionally, the tolerance of some patients to re-challenge with checkpoint inhibitor therapies suggests that RRP is not simply the result of additive toxicities from these two therapies. These findings have led some to argue that recall reactions are non-immune inflammatory idiosyncratic drug hypersensitivities caused by RT-induced reprogramming of the inflammatory pathway in treated tissues (2). To date, however, there is no established mechanism for radiation recall.

Three recent publications have described a total of 4 cases of patients with primary lung cancers developing RRP following therapy with nivolumab (3 patients) or pembrolizumab (1 patient). In one case (14), a patient developed RRP both at the site of RT for her primary lung adenocarcinoma, and at the site of prior RT for breast cancer on the contralateral lung. Similarly to our patients, all of these patients were successfully treated with oral steroids, and one patient was reported to have tolerated a re-challenge with nivolumab without recurrence of RRP.

This is the largest single-institution immunotherapy-related RRP case series. Upon presentation with RRP, these patients had very focal/asymmetric findings (inconsistent with immunotherapy-related pneumonitis) corresponding closely with the distribution of prior radiation arising acutely post treatment with the offending agent(s), and radiographic (and symptomatic for patients 1 and 3) signs showed marked improvement following treatment with steroids. Dose in the regions demonstrating exudative changes during the recall pneumonitis ranged from 12.5 to 80 Gy (**Table 1**). Interestingly, all of our patients developed symptoms while on dual-agent immunotherapy (one of whom was receiving a novel experimental immunotherapeutic on a clinical trial); patients 1 and 2 had both tolerated immunotherapy(ies) (patient 1: nivolumab and pembrolizumab, patient 2: and an experimental agent with durvalumab, as well as pembrolizumab) prior to the treatment course that is believed to have triggered the RRP. Similarly to the literature on radiation recall reactions more generally, the only patient (patient 1) to trial a re-challenge of immunotherapy did so without a recurrence of RRP. If future studies find that patients on dual-agent immunotherapy have a higher propensity towards RRP, an interesting question remains of whether this is a synergistic toxic reaction or simply an additive effect with each agent contributing a minute increased probability of a recall reaction.

**Table 1:** Dosimetric parameters including Max, Mean, V5, V10 radiation doses to the lung and heart in treated patients.

	Bilateral Lung Max Dose (tGy)	Bilateral Lung Mean Dose (tGy)	Right Lung Mean Dose (tGy)	Left Lung Mean Dose (tGy)	Lung VS (%)	Lung V20 (%)	Heart Mean Dose (cGy)
Patient 1	8100	1803	2452	1303	86.4	34.4	2440
Patient 2	5845	1134	1014	1280	69.5	10.7	819
Patient 3	3327	795	447	1293	31.1	20.8	631

There are several limitations to this case series: our patients presented with a variety of primary cancers at different stages, and received distinct systemic therapies and sequencing of radiation therapy regimens. Patient 2, for example, received radiation therapy in two stages, first with palliative intent and subsequently for consolidation as his goals of care evolved. This limits this study to hypothesis generation, and increasing awareness of the potential of recall reactions in patients treated with immunotherapeutics. Additionally, with only 3 patients, it is difficult to identify if there is a temporal relationship between radiation dose and the grade of RRP. Furthermore, there remains the possibility that these reactions may represent a ‘recall like-reaction’ due to overlapping toxicity and increased risk of pneumonitis from immunotherapy and radiation therapy, or where immunotherapy could be impacting the timing or induction of a conventional radiation pneumonitis. Nevertheless, these cases serve to highlight the potential for radiation recall reactions in the setting of immunotherapy.

As immunotherapeutics have advanced to clinical and community practice, there has been continued monitoring of their toxicities and adverse effects (19), particularly with rare outcomes unlikely to manifest in smaller and controlled clinical trial populations. In each case presented here, patients developed acute radiographic changes consistent with pneumonitis within prior radiation fields months or years after having concluded treatment. In summary, these cases together with other cases from the literature suggest that radiation recall reactions and radiation recall pneumonitis can be associated with immunotherapies. Monitoring for recall pneumonitis and further investigation of the mechanisms underlying radiation recall reactions is warranted.

## Data Availability Statement

The raw data supporting the conclusions of this article will be made available by the authors, without undue reservation.

## Ethics Statement

Patients were enrolled on UCSD IRB approved studies and provided informed consent for use of data and publication.

## Author Contributions

PR, WS, MC, SK, and ABS contributed to conception and design of the study. AS, JM, JH-G, AB, and JR managed and treated patients involved in this study. PR, WS, and MC performed analysis of patient data. PR wrote the first draft of the manuscript. PR, WS, and AS wrote sections of the manuscript. All authors contributed to the article and approved the submitted version.

## Funding

This work was supported in part by 1KL2TR001444 and a grant from the San Diego Center for Precision Immunotherapy (SDCPI).

## Conflict of Interest

AS reports research funding and honoraria from Pfizer and Varian Medical Systems, and consultant fees from AstraZeneca and Merck. ABS is the scientific founder and has an equity interest in Toragen Inc. outside the submitted work. The terms of this arrangement have been reviewed and approved by the University of California, San Diego in accordance with its conflict of interest policies.

The remaining authors declare that the research was conducted in the absence of any commercial or financial relationships that could be construed as a potential conflict of interest.

## Abbreviations

RRP, radiation recall pneumonitis; RT, radiation therapy; IMRT, intensity-modulated radiation therapy; TURBT, transurethral resection of bladder tumor.
